# Telomeres in aging and disease: lessons from zebrafish

**DOI:** 10.1242/dmm.025130

**Published:** 2016-07-01

**Authors:** Madalena C. Carneiro, Inês Pimenta de Castro, Miguel Godinho Ferreira

**Affiliations:** Instituto Gulbenkian de Ciência, Oeiras, Portugal

**Keywords:** Aging, Cancer, Disease, Telomerase, Telomeres, Zebrafish

## Abstract

Age is the highest risk factor for some of the most prevalent human diseases, including cancer. Telomere shortening is thought to play a central role in the aging process in humans. The link between telomeres and aging is highlighted by the fact that genetic diseases causing telomerase deficiency are associated with premature aging and increased risk of cancer. For the last two decades, this link has been mostly investigated using mice that have long telomeres. However, zebrafish has recently emerged as a powerful and complementary model system to study telomere biology. Zebrafish possess human-like short telomeres that progressively decline with age, reaching lengths in old age that are observed when telomerase is mutated. The extensive characterization of its well-conserved molecular and cellular physiology makes this vertebrate an excellent model to unravel the underlying relationship between telomere shortening, tissue regeneration, aging and disease. In this Review, we explore the advantages of using zebrafish in telomere research and discuss the primary discoveries made in this model that have contributed to expanding our knowledge of how telomere attrition contributes to cellular senescence, organ dysfunction and disease.

## Abstract (precis)

**Summary:** In this Review, the authors explore the advantages of using zebrafish in telomere research and discuss the primary discoveries made in this model that have contributed to expanding our knowledge of how telomere attrition contributes to cellular senescence, organ dysfunction and disease.

## Introduction

The ends of eukaryotic chromosomes are capped by telomeres, which are composed of repeated hexanucleotide DNA sequences [(TTAGGG)*n*] averaging from 5 to 15 kb in humans (Moyzis et al., 1988) and an associated protein complex known as ‘shelterin’ (de Lange, 2005). Telomeres are crucial for genome stability: they prevent chromosome ends from engaging in illegitimate repair and ensure their maintenance by recruiting the enzyme telomerase, a reverse transcriptase that elongates telomeres (de Lange, 2005).

In humans, telomerase is found active particularly in germ cells and certain adult stem cells, and can be transiently upregulated by cells of the immune system (Hiyama and Hiyama, 2007; Hodes et al., 2002; Kim et al., 1994; Weng et al., 1997). In contrast, the majority of differentiated somatic cells have no detectable levels of telomerase (Kim et al., 1994). Consequently, telomeres shorten with each round of cell division and with aging (Harley et al., 1990). Cell turnover, however, is not sufficient to predict how fast tissues lose telomere sequences (Daniali et al., 2013). Accordingly, alternative mechanisms have been proposed to contribute to telomere shortening, including post-replicative processing by exonucleases (such as Apollo and Exo1) (Chai et al., 2006; Wu et al., 2012) and oxidative stress (Oikawa et al., 2001; Passos et al., 2007; Saretzki et al., 2003).

Telomeres that have been shortened to critical lengths are recognized as DNA double-strand breaks (DSBs) and trigger mechanisms constituting DNA-damage responses (DDRs) that culminate in a specific type of cell-cycle arrest, designated by Hayflick as ‘replicative senescence’ (d'Adda di Fagagna et al., 2003; Hayflick and Moorhead, 1961; Olovnikov, 1996; van Steensel et al., 1998). Senescence induced by short telomeres can lead to both favourable and deleterious consequences. On the positive side, this phenomenon can avert the indefinite proliferation of malignant tumour cells, and thus prevent the development of cancer (Kim et al., 1994). On the negative side, it limits the function of stem cells that are necessary for tissue regeneration, potentially contributing to the loss of tissue homeostasis observed in aging (Aubert and Lansdorp, 2008; Campisi, 2014).

A prominent role for telomeres in aging has been supported by studies showing that mutations in genes crucial for telomere maintenance cause degenerative disorders that result in premature-aging symptoms (progeria-type diseases dubbed ‘telomeropathies’) (Armanios and Blackburn, 2012; Carrero et al., 2016). An example of a multisystem disorder caused by defective telomere maintenance is dyskeratosis congenita (DC). Individuals with DC often carry mutations in *TERT* and *TERC*, which encode the catalytic and RNA subunits of telomerase, respectively. Other genes that have been implicated in the disease include *TIN2* (TERF1-interacting nuclear factor 2), which encodes a component of shelterin, and genes involved in the biogenesis and trafficking of telomerase, including *DKC1* (dyskerin; dyskeratosis congenita 1), *NOP10* (nucleolar protein 10) and *TCAB1* (telomerase Cajal body protein 1) (Armanios et al., 2005; Heiss et al., 1998; Marrone et al., 2007; Trahan et al., 2010; Vulliamy et al., 2001; Vulliamy and Dokal, 2008; Walne et al., 2007; Zhong et al., 2011). DC individuals have much shorter telomeres than their unaffected relatives and die prematurely, presenting characteristic dysfunctional phenotypes in their first decade of life, including nail dystrophy, oral leukopathies and hyperpigmentation of the skin (Kirwan and Dokal, 2009). Other characteristics reminiscent of aging can develop later on, such as premature greying of the hair, hair loss (alopecia), a condition affecting teeth known as taurodontism, osteoporosis and cancer (Armanios, 2009). The majority of affected individuals die from bone-marrow failure due to an impaired renewal capability of hematopoietic stem cells (HSCs) (Basel-Vanagaite et al., 2008; Jacobs et al., 1984). Hoyeraal-Hreidersson syndrome (HHS) is a rare and severe variant of DC. In addition to DC symptoms, HHS is clinically characterized by cerebellar hypoplasia and microcephaly (Aalfs et al., 1995). Other exceptionally rare variations of DC include Revesz syndrome and Coats plus syndrome (Ramasubramanian and Shields, 2012; Scheinfeld et al., 2007). Interestingly, these disorders exhibit a pattern of genetic anticipation, in which later generations of carriers have shorter telomeres and suffer from an earlier onset of disease with aggravated symptoms (Holohan et al., 2014). *TERT* heterozygote carriers can express some form of DC and even wild-type children inherit shorter telomeres (than average) from their parents (Chiang et al., 2010). The reason why these children would inherit and maintain shorter telomeres in the presence of telomerase remains unclear.

To complement studies of humans with DC, late-generation telomerase-knockout mice (obtained by incrossing telomerase mutants for several generations, typically three or four) have been used. These mice provide a crucial laboratory tool to assess how telomere shortening promotes aging (Blasco et al., 1997; Rudolph et al., 1999). However, these mice fail to demonstrate full penetrance of DC symptoms, possibly owing to the fundamental differences in telomere length, cell immortalization and entry into senescence that distinguish mouse cells from human cells (Wright and Shay, 2000). This has fuelled the characterization of alternative telomerase-deficient vertebrate animals to more effectively bridge the gap between model organisms and humans in the study of telomere biology and aging. This Review offers a synthesis of the primary discoveries made in zebrafish models that have furthered our understanding of how short telomeres or the absence of telomerase can contribute to aging (from cellular senescence to tissue dysfunction) and disease (DC and cancer). We discuss the similarities between zebrafish telomere biology and mammalian (mouse and human) telomere biology. Finally, we raise awareness of questions that remain unsolved in the telomere-aging-disease triangle, in particular how this interplay is mediated at the molecular level and highlight the advantageous features of zebrafish – such as rapid development and ease of drug screening – that could help to address these questions in the near future.

## Zebrafish telomeres in aging – why study them?

Most short-lived rodent species die before telomeres reach the lengths found in human senescent cells (Flores et al., 2008; Gomes et al., 2011; Harley et al., 1990). The common lab mouse, which has been the primary model to date for studying how telomere shortening impacts organismal homeostasis, has telomeres ranging from 20-150 kb in length (Kipling and Cooke, 1990). Telomeres in mice are thus four- to ten-times bigger than telomeres in humans, whose average telomeres generally range from 5-15 kb (Moyzis et al., 1988; Wright and Shay, 2000). Strikingly, telomerase-deficient lab mice are viable through several generations of incrossing (mating of animals that are homozygous for the *TERT* or *TERC* loci). Previous studies showed that only later generations displayed severe disease phenotypes (e.g. premature death, infertility, intestinal atrophy, bone-marrow failure) (Blasco et al., 1997; Lee et al., 1998). This contrasts with the immediate (first generation) tissue-dysfunction phenotypes and decreased lifespan of humans carrying mutations in telomerase genes. One single study found, however, that the first generation (G1) of inbred *Terc^−/−^* mice already exhibited reductions in medium and maximum survival (García-Cao et al., 2006). It remains unclear whether the discrepancies found between these findings and those reported in earlier studies involving telomerase-knockout mice are related to strain differences.

Thus, there is a high demand for the development of alternative vertebrate models that, like humans, require telomerase for normal lifespan and tissue homeostasis. In this regard, two short-lived fish species with human-like telomeres have emerged as promising complementary vertebrate models: zebrafish (5-15 kb telomeres) and the GRZ killifish strain (6-8 kb telomeres) (Alcaraz-Pérez et al., 2014; Anchelin et al., 2013; Bednarek et al., 2015; Carneiro et al., 2016; Harel et al., 2015; Henriques et al., 2013; Imamura et al., 2008; Kim et al., 2016).

The GRZ killifish inbred strain offers a great advantage in studies of aging: of all vertebrate models bred in laboratory conditions, it has the shortest natural lifespan, with a maximum of 6 months (Harel et al., 2015). However, unlike in humans, telomere shortening with age is not observed in the GRZ strain (Hartmann et al., 2009). In addition, although telomerase deficiency in killifish causes premature tissue dysfunction, including gut villi atrophy, infertility, loss of blood cellularity and epithelial adenomatous changes, it appears not to influence the lifespan of the organism, nor embryo telomere length, in the first generation (Harel et al., 2015). Crucially, it remains to be shown whether the tissue degeneration observed in telomerase killifish mutants is a consequence of telomere shortening during adulthood. Future studies should also evaluate the possibility of using other killifish strains, such as the wild-derived strain MZM-0403 (a short-lived strain with human-like telomeres that shorten with age), as alternative models for telomere research (Hartmann et al., 2009).

**Fig. 1.**
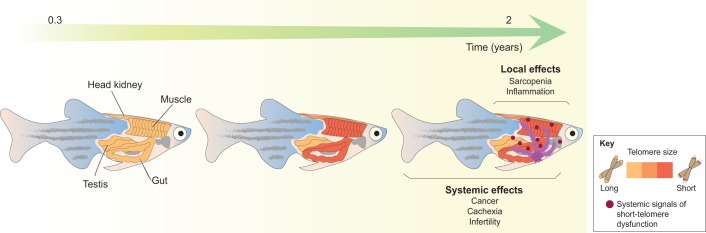
**Telomeres shorten at different rates, anticipating local and systemic tissue dysfunction in zebrafish aging.** Telomeres shorten naturally over time in specific zebrafish organs, such as the gut and muscle (but not testes), regardless of differences in proliferation rates. This shortening, together with the accumulation of local telomere damage, precludes the onset of tissue-dysfunction events in aging, including intestinal inflammation and sarcopenia. Critically short telomeres in the gut and muscle might prove to be sufficient in disrupting homeostasis in unrelated tissues, where telomeres do not shorten, by generating systemic signals (purple) of dysfunction that create a ‘disease-permissive’ environment.

The use of zebrafish, which has a maximum lifespan of 43 months (Carneiro et al., 2016), as a model to study the effects of telomerase deficiency and telomere shortening in aging, cancer and regeneration has been growing at a fast pace in the past decade (Anchelin et al., 2013; Bednarek et al., 2015; Carneiro et al., 2016; Henriques et al., 2013; Imamura et al., 2008; Wang et al., 2014). The functional domains of zebrafish telomerase are highly similar to their human counterparts [N-terminus, telomerase RNA (TR)-binding site and reverse transcriptase (RT) motifs] (Imamura et al., 2008; Lau et al., 2008). As in humans, the zebrafish telomerase promoter is activated by Myc and NF-κB (Lau et al., 2008). Telomerase expression, although detected in most zebrafish tissues, declines with age (Anchelin et al., 2011; Lau et al., 2008). Consequently, as in humans, telomeres also shorten significantly over time in tissues such as blood and muscle (Anchelin et al., 2011; Carneiro et al., 2016). Three interesting aspects of telomere shortening in zebrafish further substantiate that this organism is an effective model to study human telomere biology:

1. As reported for humans (Daniali et al., 2013), telomere shortening occurs both in high-turnover (e.g. gut) and low-turnover (e.g. muscle) organs in zebrafish, regardless of differences in proliferation rates (Carneiro et al., 2016). What determines shortening in tissues with lower proliferation rates is unknown, but causality between higher reactive oxygen species (ROS) levels and telomere shortening remains to be tested. ROS are known to cause genotoxic damage particularly in G-rich DNA regions, including telomeres (Henle et al., 1999; Oikawa et al., 2001), which could result in their attrition.
2. In zebrafish gut and muscle tissue, pronounced telomere erosion occurs within the first 1.5 years, after which no significant shortening can be detected. In humans, a similar trend of accentuated shortening during puberty followed by stabilization in length at later ages has been described (Aubert et al., 2012; Rufer et al., 1999; Sidorov et al., 2009) and could reflect the elimination of cells with extremely short telomeres, possibly via apoptosis.
3. The accumulation of short telomeres and of damage at telomeres over time in zebrafish anticipates the onset of tissue-specific phenotypes of aging, such as intestinal inflammation, and of aging-associated diseases, such as cachexia and, surprisingly, cancer (contrary to several oncogene-driven telomerase-knockout models) (Carneiro et al., 2016) (Fig. 1).

Altogether, these studies strongly support the hypothesis that, similarly to in humans but in contrast to in GRZ killifish, telomere shortening acts as a major contributor to the increase in DNA damage, tissue dysfunction and disease observed in zebrafish aging (Fig. 1). Accordingly, many larval and adult models have emerged to directly explore how these variables are interconnected. These are reviewed in the next sections and some provocative questions of where telomere research in zebrafish could lead us are raised.

## A model of vertebrate accelerated aging: the telomerase mutant zebrafish

The crucial need to evaluate how telomere shortening regulates tissue homeostasis in vertebrates with human-like telomeres has led to efforts to characterize the telomerase-deficient zebrafish strain *tert^hu3430^*^/*hu3430*^ (Anchelin et al., 2013; Henriques et al., 2013) (Table 1). First-generation *tert^hu3430^*^/*hu3430*^ zebrafish, hereafter referred to as *tert^−/−^*, have shorter telomeres than wild-type zebrafish and die prematurely (Anchelin et al., 2013; Henriques et al., 2013). *tert^−/−^* zebrafish develop several degenerative phenotypes. Homeostasis is disrupted in *tert^−/−^* highly proliferative tissues (e.g. testis and gut), resulting in infertility, gastrointestinal atrophy and inflammation. Low-proliferation tissues, such as muscle and eye, also display dysfunctional phenotypes, including sarcopenia (muscle) and retinal atrophy (eye) (Anchelin et al., 2013; Henriques et al., 2013). Strikingly, the majority of these tissue-dysfunction events tightly phenocopy those that occur during natural zebrafish aging (Carneiro et al., 2016). In addition, *tert^−/−^* zebrafish exhibit an accelerated onset of several age-related diseases, such as cachexia, gas-bladder infection and cancer (discussed below). These studies substantiate that the telomerase mutant zebrafish, an organism with artificially shortened telomeres, is an effective model to study the responses elicited by natural telomere erosion in physiological aging.

**Table 1.**
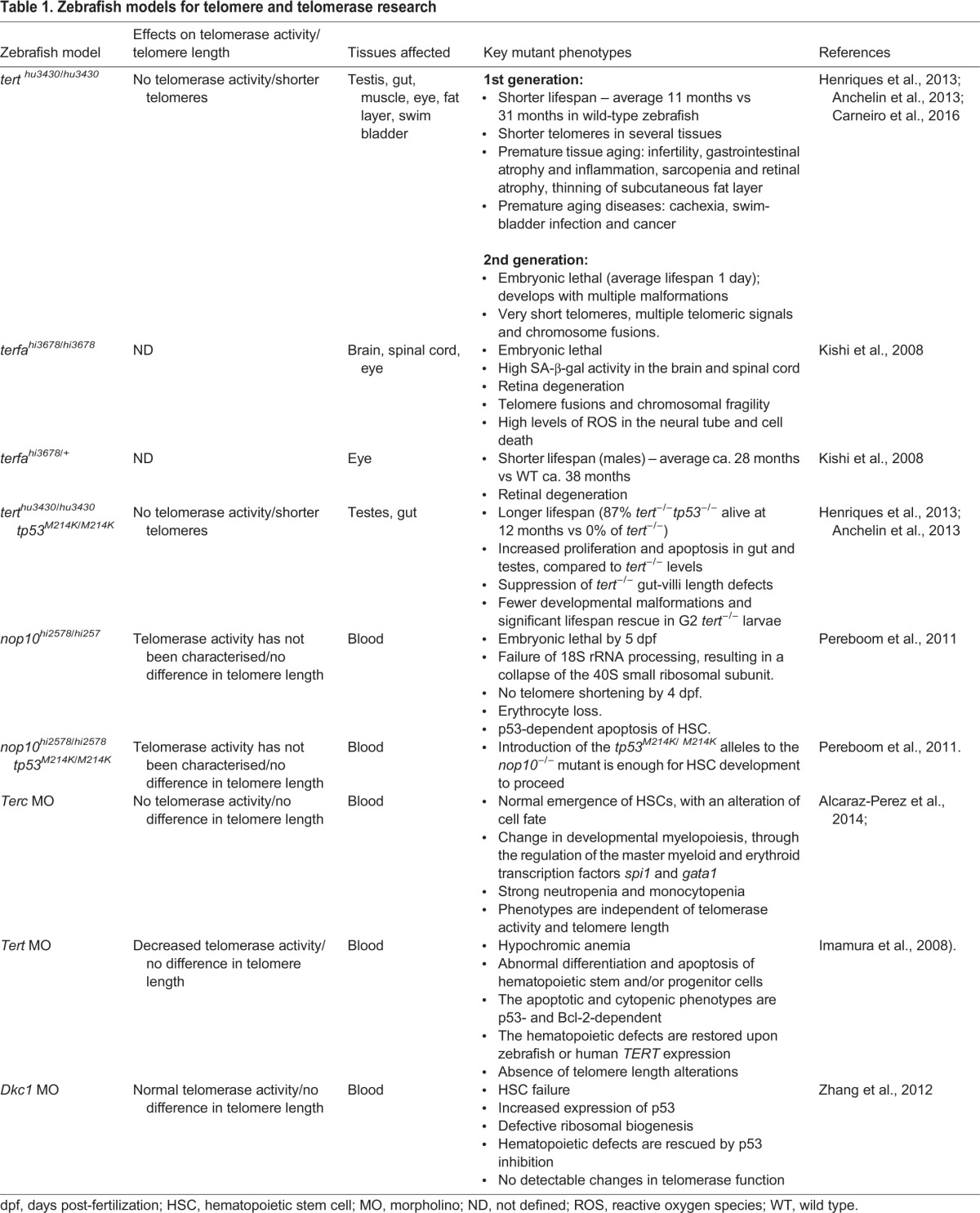
**Zebrafish models for telomere and telomerase research**

As in mammals, telomerase dosage seems to be a crucial factor for zebrafish homeostasis. This is underscored by the observation that more than 50% of a *tert^−/−^* incross progeny (second-generation *tert^−/−^* mutants or G2 *tert^−/−^*) die within the first week of life (Anchelin et al., 2013), a phenomenon that can be partially rescued by restoring telomerase activity (Anchelin et al., 2013). The severity of developmental defects found in G2 *tert^−/−^* zebrafish is proportional to the amount of critically short telomeres, measured as ‘telomere free-ends’ (Anchelin et al., 2013). Thus, telomerase deficiency causes a disease anticipation phenomenon in zebrafish, reproducing what is typically observed in human telomeropathies.

Further supporting a vital role for telomeres in zebrafish homeostasis, mutants for the telomere repeat binding factor 2 (Terf2 in mammals; Terfa in zebrafish), *terfa^hi3678/hi3678^*, are embryonically lethal (Table 1). In addition, these embryos demonstrate premature retinal neurodegeneration and senescence in the brain and spinal cord (Kishi et al., 2008). Adult *terfa* heterozygotes, although viable, exhibit signs of premature retinal degeneration and have a shorter lifespan compared with wild type (Kishi et al., 2008) (Table 1).

Many exciting questions follow these ground-breaking studies using adult zebrafish models, perhaps the most pressing being whether telomerase-activating therapeutics can delay zebrafish aging. Single telomerase gene therapy treatments using recombinant non-integrative adeno-associated viruses (AAVs) seem to be sufficient to delay the incidence of aging-associated pathologies and extend wild-type mouse lifespan, an animal with comparatively long telomeres (Bernardes de Jesus et al., 2012). Is whole-body telomerase overexpression sufficient to delay aging in zebrafish, an animal with human-like telomeres? If targeted to specific tissues where telomeres shorten over time, is telomerase overexpression sufficient to prevent local and systemic damage in aging? Is there an optimal therapeutic time window before age-associated defects become irreversible? Answers to these questions will provide crucial clues for the development of telomerase-based rejuvenation therapies.

## The complex interplay between telomeres, telomerase and cancer

Telomere shortening commits cells to growth arrest after a certain number of divisions – a mechanism that acts as a robust suppressor of tumour growth (Harley et al., 1990; Hayflick and Moorhead, 1961). Cells with short telomeres, however, can find ways to bypass replicative senescence, such as acquiring a mutation in the cell-cycle regulator p53 that favours continuing proliferation (Chin et al., 1999). Resumed proliferation ramps up chromosomal instability (‘crisis’), resulting in massive death mediated by mitotic telomere deprotection – where telomeres can no longer be distinguished from DNA damage – and chromosome fusions (Chin et al., 1999; Hayashi et al., 2015). Such events favour the selection of mutations that promote activation of telomere maintenance programs that facilitate sustained proliferation – 90% of human cancers achieve this by reactivating telomerase (Kim et al., 1994; Meyerson et al., 1997; Shay and Bacchetti, 1997). Thus, although telomere shortening might have evolved to keep cancer at bay, it also promotes the selection of unstable cells that can effectively bypass tumour-suppressor checkpoints (Artandi et al., 2000).

Surprisingly, telomerase activity is not rate-limiting for zebrafish tumorigenesis: *tert^−/−^* mutants develop spontaneous tumours at a similar frequency as wild-type animals (Carneiro et al., 2016). Similar to other aging-related diseases, cancer emerges prematurely in *tert^−/−^* zebrafish (as early as 4 months) bearing the characteristics of normal old age. Similar to wild-type zebrafish, *tert^−/−^* cancer has an 8% incidence, consists mainly of germ cell tumours, hematopoietic neoplasias and intestinal adenocarcinomas, and has a 40% invasion rate (Carneiro et al., 2016). It is not obvious how this reconciles with the scenario in mice, in which the effects of telomerase deficiency in tumorigenesis vary according to genetic context and p53 status (Artandi, 2002; Artandi et al., 2000; Artandi and DePinho, 2000). Indeed, late-generation telomerase-knockout mice have either higher cancer rates (Artandi et al., 2000; Blanco et al., 2007; Rudolph et al., 1999), lower cancer rates (sometimes with more initiation events) (Farazi et al., 2003; González-Suárez et al., 2000; Greenberg et al., 1999; Hu et al., 2012; Rudolph et al., 2001) or unaltered cancer rates (Argilla et al., 2004). These studies in mice indicate the need for a deepened understanding of how telomerase knockout affects cancer incidence, and the study of different oncogene-expressing zebrafish transgenic lines could meet this need.

Even if tumours arise in short-telomere *tert^−/−^* zebrafish cells, their growth and progression will expectedly require the activation of a telomere-maintenance mechanism. The (yet undemonstrated) explanation is that *tert^−/−^* zebrafish tumours are efficient in engaging mechanisms of alternative lengthening of telomeres (ALT) (Bryan et al., 1997). Several studies support that ALT is achieved by homologous recombination (HR) mechanisms at telomeres. Consistently, DNA tags inserted into telomeres are copied between chromosome ends in ALT cell lines, which are telomerase-negative, but not in telomerase-positive cells (Dunham et al., 2000). ALT is thought to occur in approximately 10% of human cancers (Shay and Bacchetti, 1997) and is more prevalent in tumours of mesenchymal origin (Lafferty-Whyte et al., 2009). Because zebrafish cancer rates are not affected by the absence of telomerase, it is tempting to speculate that ALT could constitute a central pathway in telomere maintenance in tumorigenesis. In the context of *tert*+ tumors, telomerase might simply provide a more stable (rather than a more common) solution to promote genome stability and outcompete ALT-based mechanisms. Consistent with this idea, recent studies show that ALT telomeres promote genome instability by recombining chromosome ends with interstitial regions (Marzec et al., 2015).

Characterizing ALT is therefore of crucial importance for the study of telomere dynamics in zebrafish cancer. Because the zebrafish genome lacks a clear promyelocytic leukemia (*PML*) gene (Veinotte et al., 2014), it is impossible to assess the presence of ALT by probing for characteristic complexes of promyelocytic leukemia nuclear bodies associated with telomeres, known as APBs (ALT-associated PML bodies). ALT tumours are nevertheless characterized by other features, including heterogeneous telomere length (with very short and very long sequences) (Bryan et al., 1997), and the presence of extrachromosomal telomeric DNA that forms double-stranded (t-circles) (Wang et al., 2004) and single-stranded (C- or G-circles) (Henson et al., 2009) circles. In addition, several recombination proteins are necessary for telomere maintenance in ALT cells, including the MRN complex (MRE11, RAD50 and NBS1) (Jiang et al., 2005; Zhong et al., 2007), subunits of the SMC5/6 (structural maintenance of chromosomes 5/6) complex (Potts and Yu, 2007), FEN1 (flap structure-specific endonuclease 1) (Saharia and Stewart, 2009), MUS81 (structure-specific endonuclease subunit) (Zeng et al., 2009) and FANCD2 (Fanconi anemia group D2) (Fan et al., 2009). Looking at a combination of these features will help determine whether ALT is a prevalent mechanism in zebrafish tumorigenesis. This could be of consequence if we are to consider the potential use of zebrafish for performing chemical screens for anti-ALT therapies with possible relevance to certain human cancers.

## Zebrafish models for dyskeratosis congenita

As detailed earlier, DC is a bone-marrow-failure disorder characterized by shortened telomeres, defective stem cell maintenance and highly heterogeneous phenotypes affecting predominantly tissues that require high rates of turnover, such as skin and lung epithelium and bone marrow (Kirwan and Dokal, 2009). Although the majority of individuals with DC die from bone-marrow failure, the associated increased risk of cancer also contributes to DC mortality (Alter et al., 2009).

All mutations identified to date in DC individuals are found in components of telomerase and in genes that are required for its biogenesis, or in telomere-stabilizing elements (Vulliamy and Dokal, 2008). All of these mutations lead to defects in telomere biology and affect the renewal capabilities of HSCs (Brümmendorf and Balabanov, 2006; Drummond et al., 2007). Mutations in telomerase genes (*TERC* and *TERT*) are autosomal dominant owing to telomerase haploinsufficiency and show disease anticipation associated with progressive telomere shortening (Vulliamy and Dokal, 2008). G1 *tert^−/−^* zebrafish have premature aging symptoms, with the most apparent phenotype being the sharp decline in the mean life expectancy (Anchelin et al., 2013; Henriques et al., 2013). Interestingly, *tert*^+/−^ zebrafish also have reduced longevity compared with wild type, while G2 *tert*^−/−^ fish die before the second week of life, suggesting that telomere length is essential for zebrafish lifespan (Anchelin et al., 2013) (Table 1). Thus, as in humans, telomerase haploinsufficiency in zebrafish leads to telomere shortening and reduced longevity, despite the presence of telomerase. Moreover, the decrease in lifespan in zebrafish is proportional to the degree of telomere shortening (Anchelin et al., 2013).

Surprisingly, mutations affecting *TERC* (such as G58A) are not associated with impaired telomerase activity *in vitro* (Chen and Greider, 2003), despite leading to particularly severe disease in humans (Vulliamy and Dokal, 2008; Chen and Greider, 2003). Various lines of evidence have shown that the cancer-promoting activity of TERC seems to be independent of *in vitro* telomerase activity, consistent with the hypothesis that TERC might play a non-canonical role in DC pathogenesis. For example, telomerase can promote tumorigenesis in mice independently of net telomere elongation (Blasco et al., 1996). Moreover, several groups have shown that mice with transgenic expression of *TERT* have a higher susceptibility to develop tumours in the absence of telomere length differences (Artandi et al., 2002; Canela et al., 2004; González-Suárez et al., 2001) and that this effect is dependent on the presence of TERC (Cayuela et al., 2005). These observations, together with the ability of TERC to specifically bind to 2198 sites in the human genome (Chu et al., 2011), have led to the hypothesis that TERC has non-canonical cellular functions, potentially involving the regulation of gene expression.

Non-canonical roles for telomerase have also been described in zebrafish. Genetic depletion of *terc* in zebrafish embryos [using antisense morpholino (MO)-mediated knockdown technology] resulted in dramatic loss of neutrophils and monocytes independent of telomerase activity or telomere shortening (Alcaraz-Pérez et al., 2014) (Table 1). Similarly, a study identified a role for *tert* in promoting the development of hematopoietic cells in zebrafish, through a mechanism that is independent of its telomerase activity and function in telomere lengthening (Imamura et al., 2008). The authors showed that *tert* morphant zebrafish embryos exhibit abnormal differentiation and apoptosis of hematopoietic stem and/or progenitor cells, subsequently leading to the circulation of immature blood cells and anaemia without any obvious telomere shortening (Imamura et al., 2008) (Table 1). This mirrors the low number of circulating blood cells – including red blood cells, white blood cells and platelets – observed in human DC (Kirwan and Dokal, 2009).

DC also encompasses genes involved in telomerase biogenesis. Dyskerin (DKC1), a member of the H/ACA ribonucleoprotein (RNP) complex, was the first gene discovered to be responsible for the X-linked severe form of DC. DKC1 is involved in telomerase function through its RNA subunit, which contains an H/ACA RNA motif, and integrity of this motif is essential for assembly and stability of the human telomerase RNP (Vulliamy et al., 2006). Knockdown of *dkc1* revealed a role in zebrafish hematopoiesis (Zhang et al., 2012). Downregulation of *dkc1* results in HSC failure, increased p53 expression and defective ribosomal biogenesis, all without detectable changes in telomerase activity (Zhang et al., 2012) (Table 1). Mutations in two other H/ACA RNP complex genes, *NHP2* (Vulliamy et al., 2008) and *NOP10* (Walne et al., 2007), have also been reported in DC. In line with the previously discussed zebrafish DC models, *nop10* mutant embryos also fail to form HSCs and, again, these mutants display no telomere shortening (Pereboom et al., 2011) (Table 1).

In all four of these zebrafish models, telomere lengths are not significantly altered, supporting a model for DC pathogenesis where mutations in *TERC*, *TERT*, *DKC1* and *NOP10* contribute to bone-marrow failure through a telomere-lengthening- independent mechanism. In line with these observations, some of the previously reported disease-associated human TERT alleles give rise to a near-normal telomerase enzyme activity, suggesting that these mutations cause the disease by affecting telomerase functions that are not related to its enzymatic activity (Zaug et al., 2013). These findings are in apparent contradiction with those obtained in telomerase*-*deficient mice, which exhibit defects in the haematopoietic system only when telomeres are critically short (Lee et al., 1998). The most obvious explanation is that there might be developmental and/or physiological compensations in mice that probably do not exist in humans or fish.

Despite the concordance between findings in zebrafish and studies of human clinical samples, it is important to highlight that the zebrafish studies use MOs to downregulate the expression of *tert*, *terc* and *dkc1*. Recently, the use of this technology has been questioned throughout the zebrafish community. Unfortunately, some morphant phenotypes have been proven to be due to off-target effects, suggesting that it is always preferable to target exons encoding domains that are necessary for protein function or generating segmental deletions, rather than to use MOs (Kok et al., 2015). Given the ease of use of current site-specific nuclease technologies, most notably the CRISPR systems, it will be crucial to generate new zebrafish lines, ideally carrying mutations similar to those found in humans with DC, in order to confirm the importance of the non-canonical functions of telomerase.

## How do short telomeres drive aging?

As mentioned earlier, induced telomere shortening (in telomerase mutants) is sufficient to cause a cascade of tissue dysfunctional events in both high- and low-proliferation tissues. However, the mechanistic basis underlying loss of homeostasis in the majority of these tissues is still not understood. Deciphering whether these mechanisms are also relevant in contexts of natural aging is crucial to understand the impact of short telomeres in age-associated diseases.

Telomeres shortened to critical lengths become indistinguishable from DNA double-strand breaks triggering DDRs (de Lange, 2005), which results in the activation of p53 (d'Adda di Fagagna et al., 2003; Gire et al., 2004; Guo et al., 2007). Accordingly, p53 acts as a major executioner of short-telomere-induced defects in highly proliferative tissues of both mice (Chin et al., 1999) and zebrafish (Anchelin et al., 2013; Henriques et al., 2013). Deletion of *p53* is sufficient to prevent germ-cell apoptosis and infertility in late-generation *Terc^−/−^* mice (Chin et al., 1999). However, this comes at the high cost of increased genome instability and cancer (Artandi et al., 2000; Chin et al., 1999; O'Hagan et al., 2002). Although p53 deficiency rescues premature death, cell-proliferation defects and reduced gut-villi length of *tert^−/−^* zebrafish (Anchelin et al., 2013; Henriques et al., 2013) (Table 1), its effect on tumour incidence has not been described. Most likely, *tert^−/−^ tp53^−/−^* zebrafish will display an abnormally high incidence of cancer and early onset of tumors, given that these features characterize the single *tp53^−/−^* mutants [28% of fish develop malignant peripheral-nerve-sheath tumors by 8 months (Berghmans et al., 2005)] and the single *tert^−/−^* mutants (where cancer appears as early as 4 months) (Carneiro et al., 2016). Alternatively, given that most *tert^−/−^* phenotypes related to aging are suppressed in the absence of p53, it is also conceivable that the onset of spontaneous tumours could be delayed in *tert^−/−^ tp53^−/−^* zebrafish.

**Fig. 2.**
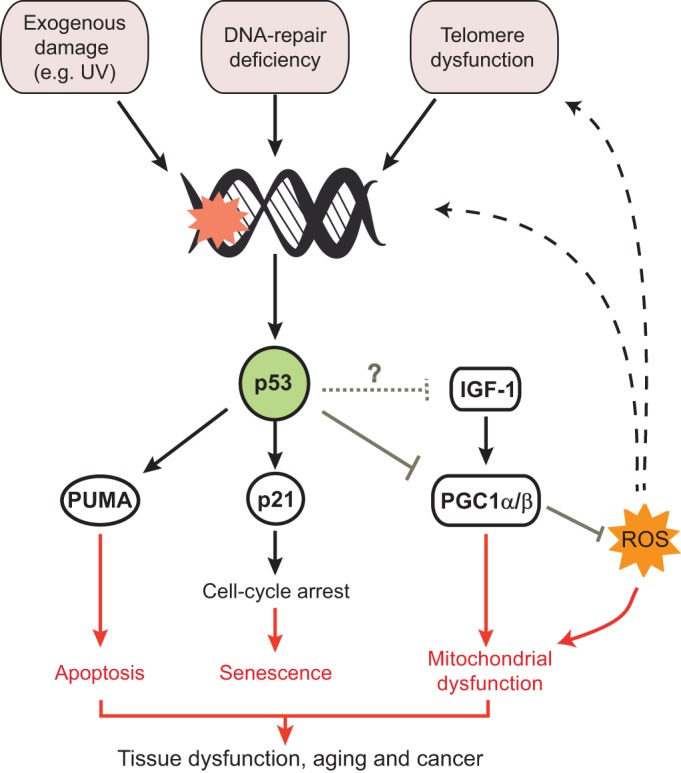
**Pathways modulated by the short-telomere–p53 axis.** Telomere dysfunction, as well as exogenous genotoxic agents and deficiencies in DNA repair, activates p53 (Chin et al., 1999), causing PUMA-mediated apoptosis (Sperka et al., 2012) and p21 cell-cycle arrest, and, consequently, cell senescence (Choudhury et al., 2007). p53 upregulation also leads to impairments in energy homeostasis and potential suppression of IGF-1 signalling, which result in repression of master regulators (such as PGC1α/β) of mitochondrial biogenesis. This leads to mitochondrial dysfunction and, consequently, increased ROS levels, which promote further damage at telomeres. ROS, reactive oxygen species. For further information and references, see the main text.

Which molecular players act downstream of p53 to disrupt tissue homeostasis in *tert^−/−^* zebrafish? A starting point to address this question would be to investigate the molecular mechanisms already described to mediate tissue dysfunction in late-generation telomerase-knockout mice (Fig. 2), namely:

Type I: activation of the cell-cycle inhibitor p21 together with p53-dependent modulator of apoptosis (PUMA). These molecules are known to limit stem cell proliferation and the regeneration capacities of high-turnover organs (e.g. intestine, hematopoietic tissue) in G3/G4 *Terc^−/−^* mice (Choudhury et al., 2007; Sperka et al., 2012).
Type II: repression of peroxisome proliferator-activated receptor gamma coactivator 1-alpha/beta (PGC1α/β), master regulators of mitochondrial biogenesis [possibly mediated by inhibition of insulin-like growth factor 1 (IGF-1) and mammalian target of rapamycin (mTOR) signalling]. Reduction of PGC1α/β lowers oxidative defence mechanisms and gluconeogenesis, resulting in an accumulation of ROS (Fig. 2). Altogether, these events have been proposed to underlie dysfunction of more quiescent organs (e.g. heart) in G3/G4 *Terc^−/−^/Tert^−/−^* mice (Missios et al., 2014; Sahin et al., 2011).

Importantly, activation of type I or II mechanisms might not be mutually exclusive; instead, these processes could occur simultaneously within an individual tissue undergoing telomere attrition. Because organs are composed of multiple cell types with a wide spectrum of proliferative profiles, this raises the intriguing question: which cells within a tissue undergoing telomere attrition invoke which mechanism? What determines the choice?

There is already compelling evidence showing that stem cells with short telomeres typically activate type I mechanisms, undergoing growth arrest and apoptosis in the intestinal, skin, brain and hematopoietic tissues (Choudhury et al., 2007; Ferrón et al., 2004; Flores et al., 2005; Rajaraman et al., 2007; Sperka et al., 2012; Wong et al., 2003). A recent study by Rudolph and colleagues further demonstrated that this occurs only in stem cells that are actively cycling (and not in those lying in a quiescent state), presumably to prevent transmission of aberrant genotoxic damage to progenitor cells (Wang et al., 2014). We still do not know how cells other than adult tissue stem cells deal with telomere-induced DDRs. Testing *in vivo* how the expression of a given gene or pathway is affected in specific telomere dysfunctional cells is a complex task. Zebrafish is an ideal model for addressing these questions, given the possibility for live visualization of the expression of different fluorescent reporters using transparent telomere dysfunctional strains (specifically, albino strains). Nevertheless, there are intrinsic limitations to the use of zebrafish – an emerging model – in this area of research: it is a non-mammalian system that either lacks specific cell markers or the reagents to detect them. Furthermore, it remains to be shown whether key metabolic processes in this cold-blooded animal are truly indistinguishable to parallel processes in humans.

## Telomerase, telomeres, senescence and tissue repair

Telomeres function as molecular clocks that keep a record of the replicative history of primary cells (Harley et al., 1990). Telomere erosion through consecutive cell divisions results in critically short telomeres and elicits replicative senescence (Hayflick and Moorhead, 1961; Wang et al., 2014; Bodnar et al., 1998). Importantly, short telomeres and senescent cells accumulate in some, but not in all, tissues in aged humans (Dimri et al., 1995), monkeys (Herbig et al., 2006) and mice (Wang et al., 2009). Since its initial description, our understanding of cellular senescence has evolved dramatically and, today, the concept of senescence has been redefined. In this section, we discuss the importance of senescent cells in an organism, their potential role in tissue regeneration and how this beneficial process can be degraded, particularly in aged tissues.

In zebrafish, telomeres shorten during aging (Carneiro et al., 2016; Anchelin et al., 2011) and upon loss of telomerase (*tert^−/−^*) (Anchelin et al., 2013; Henriques et al., 2013). In both cases, the rate of telomere decline varies between tissues (Carneiro et al., 2016). As telomeres shorten, the number of senescent cells increases in the skin (Kishi, 2004; Kishi et al., 2003), gut, testes and kidney marrow (head kidney serves as the hematopoietic tissue in zebrafish) (Carneiro et al., 2016). *terf2* mutant embryos, which have dysfunctional telomeres, also exhibit high levels of senescence-associated β-galactosidase (SA-β-gal) activity, a widely used *in vitro* marker for cellular senescence as well as of organismal aging in vertebrates (Kishi et al., 2008). Moreover, the brains of old zebrafish express high levels of *smurf2*, a gene implicated in the induction of replicative senescence (Zhang and Cohen, 2004), highlighting short telomeres as important triggers for cell senescence in this teleost (Arslan-Ergul and Adams, 2014).

As telomeres get critically short, cellular senescence is activated, and this leads to engagement of various signalling cascades that ultimately activate p53, p16^INK4a^ or both. In mammals, activated p53 induces p21, causing a temporal cell-cycle arrest through the inhibition of cyclin-E–Cdk2 (Beausejour et al., 2003). p16^INK4a^ also inhibits cell-cycle progression but does so by targeting cyclin-D–Cdk4 and cyclin-D–Cdk6 complexes (Sherr and Roberts, 1999). Both p21 and p16^INK4a^ act by preventing CDK inactivation of Rb (retinoblastoma protein), resulting in continued repression of *E2F* target genes required for S-phase onset. The relative contribution of p53, p21 or p16^INK4a^ to the initial growth arrest can vary depending on the type of stress. Their function, either alone or in combination, could ultimately result in sustained senescence.

In zebrafish, the p53-p21 pathway is known to exist with relevant functional conservation (Berghmans et al., 2005). As for the second effector pathway, the human genetic locus that encodes for p16^INK4a^ also encodes for p15^INK4b^ and a p53 stabilizer, known as p14^ARF^ (p19^ARF^ in zebrafish). Despite the crucial role of the mammalian *INK4b-ARF-INK4a* locus in tumour suppression, its counterpart in zebrafish has not yet been fully characterized. Recently, Sabaawy and colleagues identified one locus homologous to human INK4 in zebrafish, which, surprisingly, was devoid of ARF sequences (Sabaawy et al., 2006). This locus encodes a single zebrafish *ink4ab* gene, which functions to activate senescence in response to oxidative stress. Thus, zebrafish INK4ab seems to function as a tumour suppressor similar to human p15^INK4B^ and p16^INK4A^ (Davis and Sabaawy, 2013; Flaherty et al., 2015).

Although established senescence markers are lacking, most senescent cells express the tumour suppressor p16^INK4a^ (Ohtani et al., 2004), the levels of which increase with age (Krishnamurthy et al., 2004; Ressler et al., 2006). This rise of p16^INK4a^ often coincides with SA-β-gal activity (Dimri et al., 1995). Kishi and colleagues reported SA-β-gal induction during aging in zebrafish (Kishi, 2004; Kishi et al., 2003). His group has also been successful in exploiting SA-β-gal staining as a marker for organismal senescence in zebrafish larval models of premature aging (Kishi et al., 2008).

Curiously, senescent cells are still metabolically active and release a complex mixture of extracellular matrix proteases, growth factors, chemokines and cytokines [collectively known as senescence-associated secretory phenotype (SASP)] that has significant effects on the surrounding tissue microenvironment. SASP has not yet been described in zebrafish; however, in mammals, it seems to have an important role in recruiting immune and phagocytic cells, such as macrophages, and also activates the motility and proliferation of surrounding cells (Muñoz-Espín and Serrano, 2014).

Nowadays, senescence has been proposed as an alternative way of cell death. While apoptosis is a rather individual and silent process of cellular suicide, the senescent program requires the involvement of different and complex players to bring about the same result: the clearance of damaged cells (Muñoz-Espín and Serrano, 2014). Still, the ultimate goal of senescence remains unclear. If in the end, senescence is just another way to eliminate damaged cells then why not choose a more direct and faster route of apoptosis?' It has been proposed that the central role of senescent cells is to initiate a tissue-remodelling process that includes their own elimination. A particularly striking example of the role of senescence in tissue remodelling has been the recent demonstration that senescence participates in developmental processes in vertebrates (Muñoz-Espín et al., 2013; Storer et al., 2013), promotes wound healing in mice (Demaria et al., 2014) and contributes to heart regeneration in zebrafish (Bednarek et al., 2015).

**Fig. 3.**
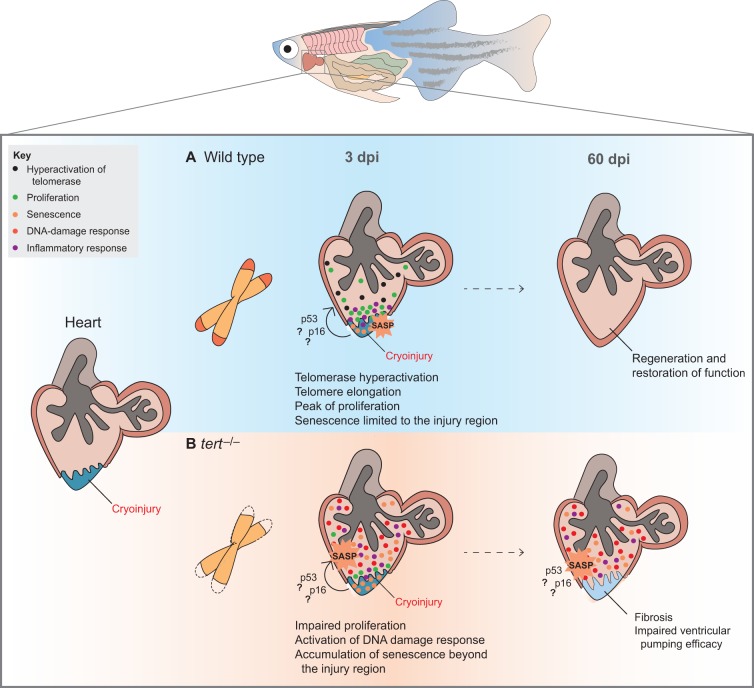
**The role of telomerase, telomeres and senescent cells in zebrafish heart regeneration.** Cryoinjury in zebrafish mimics aspects of human myocardial infarction. (A) In wild-type fish, cardiomyocyte proliferation is sharply increased in response to tissue damage, accompanied by an increase in *tert* gene expression, hyperactivation of telomerase and a transient elongation of telomeres (3 dpi). Additionally, there is an accumulation of senescence cells, limited to the injured region (3 dpi) that is cleared upon wound closure (60 dpi) (Bednarek et al., 2015). Senescent cells release growth factors and cytokines, which might activate the motility and proliferation of surrounding cells, potentiating tissue remodelling. (B) Aged cardiac tissues, modelled by the absence of telomerase (*tert^−/−^*), are not amenable to tissue remodelling, reflecting a combination of factors, such as proliferative defects, accumulation of DNA-damaged cells and increased senescence (3 dpi and 60 dpi). The difficulty in handling and clearing damaged and senescent cells might overload the tissue with the senescence-associated secretory phenotype (SASP), which potentially contributes to a persistent chronic inflammatory microenvironment that further aggravates tissue dysfunction and impairs proper wound closure (Bednarek et al., 2015).

Zebrafish has become a powerful model for investigating tissue remodelling owing to its capacity to completely regenerate several organ injuries, including brain, spinal cord, retina, heart and fins, even at mature adult stages (Gemberling et al., 2013). This ability to regenerate declines with age (Kishi et al., 2009) and, in some organs, is heavily dependent on telomerase activity (Bednarek et al., 2015; Elmore et al., 2008) (Fig. 3). Bednarek and colleagues have recently shown that ventricular cryoinjury, a process that induces massive cell death similar to that observed in a leading cause of mortality and morbidity in humans – myocardial infarction – leads to telomerase hyperactivation in cardiomyocytes, accompanied by a sharp peak of proliferation and a transient elongation of telomeres (Fig. 3A). Strikingly, SA-β-gal signalling was induced upon cryoinjury, denoting an initial and transient accumulation of senescent cells (Bednarek et al., 2015) (Fig. 3A). However, senescence was limited to the injured region and easily cleared upon wound closure. These observations are in line with studies in mice (Demaria et al., 2014), supporting the hypothesis that, in an *in vivo* context, cellular senescence might contribute to tissue remodelling.

The full benefits of senescence are achieved when the process includes the clearance of the senescent cells, thereby restoring the pre-damage status of the tissue. However, in chronic pathological situations such as aging (as modelled by *tert*^−/−^ fish), cardiac regeneration is impaired and a fibrotic scar remains. This inability to regenerate is primarily due to a strong inhibition of the proliferative response and an accumulation of senescent cells that becomes persistent, and these cells even extend beyond the injured area, further aggravating tissue dysfunction (Bednarek et al., 2015) (Fig. 3B). The difficulty in handling and clearing damaged and senescent cells could overload the tissue with SASP. This effect results in a persistent chronic inflammatory microenvironment that further aggravates tissue dysfunction and impairs proper regeneration. This process might not be applicable to other types of aged tissues, but constitutes a clear example of how short telomeres and cellular senescence can contribute to age-related defects in tissue regeneration.

Senescence is a double-edged sword, beneficial when it is transient and easily handled but pathological when chronic and unresolved. So, what makes an aged tissue more prone to the accumulation of senescent cells? On the one hand, clearance of senescent cells by the immune system might become impaired with aging, leading to a net accumulation of senescent cells that further aggravate tissue dysfunction via the SASP. On the other hand, senescence might not only affect differentiated cells but also stem and progenitor cells, thus limiting the regenerative capacity of tissues.

Modern society is extremely interested in finding ways to extend human healthy lifespan. There are ongoing pharmacological tests and biological therapies to prevent telomere shortening and accumulation of senescent cells during aging. The impact this will have on human health and disease is currently unknown, although it will likely reveal new biological phenomena. If shortening of telomeres can be prevented and/or senescent cells can be eliminated in human tissues, will this simply delay the very familiar aging phenomenon, or will new types of pathology emerge? These nuances and complexities demand further investigation in order to guide potential new therapeutic options.

## Concluding remarks

Although telomere shortening is considered a primary culprit of human aging, many questions remain unresolved. How do short telomeres disrupt homeostasis in particular tissues over time? Which precise molecular mechanisms mediate such tissue dysfunction events in aging and premature-aging syndromes such as DC? Addressing these issues has long been hampered by the lack of a vertebrate laboratory model that recaps crucial human features, including a 5-15 kb telomere length range and the immediate onset of degenerative phenotypes upon removal of telomerase.

Ground-breaking discoveries establishing zebrafish as a powerful model for the study of vertebrate telomeres have now filled this need. Because zebrafish has human-like telomeres, deletion of telomerase causes an acceleration of aging-associated diseases and tissue dysfunction events that are observed in the first mutant generation (Anchelin et al., 2013; Carneiro et al., 2016; Henriques et al., 2013). These not only phenocopy what is observed in human aging, but also reproduce the typical ‘anticipation phenomenon’ reported in humans with DC (younger generations have an earlier disease onset) (Anchelin et al., 2013; Henriques et al., 2013). Thus, phenotypes and molecular mechanisms are both highly conserved from mammalian to zebrafish in the context of telomere dysfunction, rendering this teleost a promising model for telomere research in aging.

From testing the impact of telomerase therapeutics targeted to specific tissues to identifying the molecular mechanisms that mediate short-telomere-induced dysfunction in aging, several challenges lie ahead in the field of telomere research using zebrafish. The unique characteristics of this organism have already proven useful for the identification of important new links between tissue repair, telomerase activation and cellular senescence. Defining how telomere dysfunction is interconnected to other hallmarks of aging is bound to revolutionize the future of aging therapeutics.
